# Socioeconomic status and ECMO outcomes in severe ARDS

**DOI:** 10.1016/j.aicoj.2025.100012

**Published:** 2026-01-16

**Authors:** Diane Naouri, Matthieu Jamme, Alain Combes, Antoine Kimmoun, Matthieu Schmidt

**Affiliations:** a Direction de la recherche, des études, de l’évaluation et des statistiques (DREES), Ministère du Travail, de la Santé, des Solidarités et de la Famille, Paris, France; b Service de Médecine intensive – Réanimation, Hôpital privé de l’ouest parisien, Ramsay Général de Santé, Trappes, France; c Sorbonne Université, Service de Médecine intensive – Réanimation, Institut de cardiologie, Assistance Publique-Hôpitaux de Paris, Hôpital Pitié-Salpêtrière, Paris, France; d Univeristé de Lorraine, CHRU Nancy, Service de Médecine intensive – Réanimation, Nancy, France

**Keywords:** Extracorporeal membrane oxygenation, Acute respiratory distress syndrome, COVID-19, Influenza, Social deprivation

## Abstract

**Background:**

Socioeconomic inequalities have been associated with adverse outcomes in critically ill COVID-19 patients. Whether these disparities extend to the most severe ARDS patients treated with ECMO, regardless of etiology, remains uncertain. We aimed to compare the socioeconomic profiles, management, and outcomes of COVID-19 ARDS patients on ECMO with those treated for ARDS due to other causes, using the nationwide French healthcare database.

**Results:**

From March 2015 to December 2021, 1722 adults received ECMO for acute respiratory failure: 1245 with COVID-19, 107 with influenza, and 370 with other causes. Overall, 27% lived in the most deprived neighborhoods, with consistent overrepresentation across etiologies (26.8% COVID-19, 29% influenza, 25.3% other) compared to less deprived neighborhoods (p = 0.039). In-hospital mortality was 56% in COVID-19, 48% in influenza, and 60% in other causes (p = 0.080). Median ICU stay was longest in COVID-19 survivors (56 [36–78] days), who also required longer ECMO support and experienced more complications. Independent predictors of in-hospital death included older age and need for renal replacement therapy at ECMO initiation, while socioeconomic deprivation was not associated with outcomes. After adjustment, mortality was higher in non-COVID-19, non-influenza patients compared with influenza (Odds ratio 1.70, 95% confidence interval [1.03–2.81]).

**Conclusions:**

Severe ARDS requiring ECMO disproportionately affected patients from socioeconomically deprived areas, irrespective of etiology. However, deprivation was not linked to worse outcomes.

## Introduction

COVID-19 has been an unprecedented modern pandemic, infecting over 777 million people and claiming nearly seven million lives worldwide in just six years [1]. During its first wave, 5%–10% of hospitalized patients required intensive care, and around 10% of these critically ill individuals received extracorporeal membrane oxygenation (ECMO) [2]. Although no randomized trial has confirmed its survival benefit, ECMO remains a recommended therapy, endorsed by critical care experts and the European Society of Critical Care, for patients with the most severe forms of COVID-19 acute respiratory distress syndrome (ARDS) unresponsive to optimal medical management, similar to how it is used for ARDS caused by other infections like influenza [3,4]. Comparisons with seasonal influenza epidemics reveal the heavier toll of COVID-19: higher ICU admission rates (16% vs. 10%), more frequent mechanical ventilation (71% vs. 61%), and greater in-hospital mortality (27% vs. 18%) [5,6]. Despite extensive research on the pre-ECMO and ICU phases, little is known about how ARDS patients on ECMO for COVID-19 compare with those whose ARDS results from other infectious or non-infectious causes, especially across the full care continuum, from ECMO selection to long-term recovery. Social determinants of health add another layer of complexity: densely populated housing has been linked to an increased risk of COVID-19 hospitalization, and precarious living conditions to higher in-hospital mortality [7,8]. Whether these disparities also influence the outcomes of the sickest ARDS patients on ECMO, regardless of etiology, remains unknown. To address this gap, we utilized the nationwide French administrative healthcare database to examine the socioeconomic profiles and full healthcare trajectories of COVID-19 ARDS patients treated with ECMO, compared with those receiving ECMO for ARDS related to other causes.

## Patients & methods

### Study design

We conducted a retrospective cohort study using the French administrative healthcare database, the *Système National des Données de Santé* (SNDS). The SNDS includes detailed information on outpatient care (such as medical consultations, paramedical interventions, and reimbursed drug distributions) and hospital data from the *Programme de Médicalisation des Systèmes d’Information* (PMSI), including admissions, length of stay, ICD-10 diagnostic codes (both primary and secondary diagnoses), and medical procedures [9]. Records are securely linked via a unique personal identification number. Access to and use of the SNDS database are governed by French government regulations, as outlined in the decree dated March 22, 2017 [10]. As part of its public health and statistical duties, the French Ministry of Health's *Department for Research, Studies, Assessment, and Statistics* has ongoing access to the SNDS. Additionally, an official public webpage provides information about the reuse of SNDS data and individual rights under the European General Data Protection Regulation No. UE 2016/679, which took effect on April 27, 2016 [11].

### Study population

We included adults (≥18 years) residing in metropolitan France who were admitted to the ICU between March 1, 2020, and December 31, 2021, with a complete hospital course available and at least one ICD-10 diagnosis code for COVID-19, as previously described [12]. Among these patients, those who received ECMO for respiratory failure were identified based on procedure codes from the French Common Classification of Medical Procedures (CCAM) [13], specifically CCAM code GLJF010. For the non-COVID-19 cohort, we included all adult patients (≥18 years) admitted to French ICUs between 2015 and 2019 with a primary diagnosis of influenza (ICD-10 codes J09, J10, J11), pneumonia (ICD-10 codes J12–J18), or ARDS (ICD-10 code J80), who also required ECMO support. Patients were subsequently stratified into two groups based on primary and associated diagnoses: an influenza group and a non-COVID-19 or influenza group.

### Data collection

Demographic and clinical data, including age, sex, and the Simplified Acute Physiology Score (SAPS) II [14] at ICU admission, were recorded for each inpatient stay. Relevant pre-existing comorbidities were also documented, including arterial hypertension, diabetes mellitus, cardiovascular disease, cirrhosis, malignancies (both solid organ and hematologic), and chronic kidney disease. Immunocompromised status was defined by the presence of agranulocytosis, medullary aplasia, HIV infection, malignancy requiring chemotherapy, history of solid organ transplantation, or prolonged use of corticosteroids or immunosuppressive agents (details of the ICD-10 code used are provided in the eFile 1).

During hospitalization, several clinical outcomes were assessed. The use of prone positioning, vasopressor therapy, or renal replacement therapy (RRT) before ECMO initiation was identified. The duration of hospitalization, ICU stay, ECMO support, and invasive mechanical ventilation (IMV) was recorded. In addition, the interval between IMV initiation and ECMO initiation (IMV–ECMO timing) and patient age were categorized as follows: <3 days, 3–7 days, and >7 days for IMV–ECMO timing, and ≤48 years, 49–56 years, and ≥57 years for age [15]. These classifications and cutoffs were selected based on previously demonstrated associations between these variable categories and mortality [15].

### Post-hospital discharge

For patients who survived hospitalization, data on outcomes after discharge were collected. This included transfer to post-acute care and rehabilitation services or home-based hospitalization within the first month post-discharge. Additional variables involved the need for long-term oxygen therapy, rehospitalization within one year, and new pulmonology consultations (defined as a first pulmonology visit for patients with no prior consultation in the previous year). Furthermore, the start of bêta-2 agonist therapy (for individuals without prior use in the year before hospitalization), the number of days receiving sickness allowances (compensation for income loss due to work incapacity or medical leave for those under 65), and all-cause mortality at one year were also analyzed.

### Socio-economic variables

The French Deprivation Index (FDep) is a neighborhood-level indicator of social disadvantage derived from four components: unemployment rate, proportion of blue-collar workers, high school graduation rate, and median taxable household income [16]. In this study, the FDep was used as a proxy for socioeconomic position. This ecological approach is commonly employed in public health research to examine social health inequalities when individual-level socioeconomic data are unavailable. It relies on the assumption that individuals living in socially deprived areas are more likely to experience socioeconomic disadvantage themselves. Higher FDep scores correspond to greater deprivation. Patients classified in the highest FDep quintile therefore reside in municipalities identified as highly deprived, regardless of their geographic location, and consequently have a higher likelihood of being socially disadvantaged. The geographical distribution of the FDep in France at the regional and municipal levels is presented in eFile 2. For analytical purposes, FDep values were grouped into five quintiles based on their distribution in the French population, with Q1 representing the most advantaged neighborhoods and Q5 the most disadvantaged. Each patient was assigned an FDep quintile according to their residential address. In addition, information on beneficiaries of the Couverture Maladie Universelle Complémentaire (CMU-C) was collected. The CMU-C is a French supplementary health insurance program that provides free complementary coverage to individuals with very low income. It covers out-of-pocket expenses not reimbursed by the national health insurance system, including medical consultations, medications, and hospital costs. Beneficiaries also have access to care without upfront payment. The CMU-C aims to reduce financial barriers to healthcare and ensure equitable access to essential medical services.

### Statistical analysis

We followed the STROBE (Strengthening the Reporting of Observational Studies in Epidemiology) recommendations for reporting cohort studies [17]. Patient characteristics are expressed as numbers (percentages) for categorical variables, and median [interquartile range (IQR)] for continuous variables. Categorical variables were compared using the chi-square or Fisher’s exact test, and continuous variables using the Wilcoxon test. Comparisons among the three etiology groups and across FDep quintiles were performed using the Kruskal–Wallis test. When normality was confirmed by the Shapiro–Wilk test, a one-way ANOVA was used instead. The study population and outcomes were also described according to patients’ FDep quintile.

Baseline risk factors for in-hospital death were evaluated across the entire cohort using multivariate logistic regression models. Baseline variables (i.e., those obtained before ECMO initiation) included in the multivariable model were predefined, and no variable selection was conducted. Collinearity among explanatory variables was checked using correlation matrices and the Variance Inflation Factor (VIF). All VIF values were below commonly accepted thresholds, indicating no problematic collinearity and allowing all chosen predictors to remain in the model. Model calibration was assessed with the Hosmer–Lemeshow goodness-of-fit test. Clinically plausible interaction terms, such as age × ARDS etiology and age × time from intubation to ECMO initiation, were tested but not statistically significant. Model discrimination was evaluated using the C-statistic. Since our analyses rely on medico-administrative data from hospital coding systems, variables like age and sex are consistently recorded and contain no missing data. Likewise, clinical variables such as procedures are only coded when the event occurs, inherently preventing any missingness for these indicators. Therefore, no data imputation was necessary. Odds ratios with 95% confidence intervals were estimated. All the analyses were computed at a two-sided alpha level of 5%. Statistical analyses were conducted with SAS 2017 software (SAS Institute, Cary, NC, USA).

## Results

### Study population

A total of 1722 patients receiving ECMO for acute respiratory failure were included during the study period. Among them, 1245 had COVID-19, 107 had influenza, and 370 had ARDS from other causes than COVID or influenza (Table 1).

**Table 1:** Characteristics of the study population.

	ECMO for COVID-19 (N = 1245)	ECMO for influenza (N = 107)	ECMO for other causes (N = 370)	P value
Age	56 (48−63)	56 (46−63)	57 (43−67)	0.213
⩽ 48 years	342 (27)	32 (30)	115 (31)	
49–56 years	291 (23)	22 (21)	62 (17)	
≥ 57 years	612 (49)	53 (49)	193 (52)	
Male	880 (71)	69 (64)	246 (66)	0.160
SAPS II	33 (26−44)	43 (33−59)	45 (33−59)	<.001
French Deprivation Index quintile *				
1−3	607 (57)	41 (44)	151 (52)	0.001
4−5	467 (33)	52 (56)	142 (48)	
Beneficiaries of CMU-c **	181 (16)	18 (17)	69 (21)	0.149
Comorbidities				
Arterial hypertension	463 (37)	25 (23)	48 (13)	<.001
Diabetes mellitus	310 (25)	3 (3)	4 (1)	<.001
Cardiovascular disease	110 (9)	9 (8)	20 (5)	0.103
Cirrhosis	46 (4)	1 (1)	4 (1)	0.015
Solid organ cancer	28 (2)	0 (0)	6 (2)	0.237
Hematological malignancies	19 (1)	2 (2)	7 (2)	0.869
Chronic kidney disease	30 (2)	2 (2)	2 (0.5)	0.076
Immunocompromised status	72 (6)	5 (5)	22 (6)	0.879
Time between ICU admission and ECMO, days	7 (4−12)	3 (1−7)	3 (1−9)	<.001
Time between ICU admission and IMV, days	2 (0−5)	0 (0−1)	0 (0−2)	<.001
Time between IMV and ECMO, days	4 (1−7)	1 (0−5)	1 (0−6)	<.001
Procedures before ECMO onset				
Prone positioning	1037 (83)	84 (78)	214 (58)	<.001
Vasopressors	575 (46)	66 (62)	181 (49)	0.008
Renal replacement therapy	331 (27)	11 (10)	42 (11)	<.001

CMU-C, a French supplementary health insurance program providing free complementary coverage to individuals with very low income; ECMO, extracorporeal membrane oxygenation; IMV, invasive mechanical ventilation; ICU, intensive care unit; SAPS, Simplified Acute Physiology Score.

Data are presented as median (interquartile range) or N (%).

* Data available in 1460 patients (262 missing values).

** Data available in 1567 patients (155 missing values).

The age distribution was similar across the three groups, with half of the population being older than 57 years. The SAPS II score was higher in patients receiving ECMO for influenza or other causes than in those with COVID-19 (p < 0.001). However, patients with COVID-19 more frequently had arterial hypertension or diabetes mellitus. The median time between starting IMV and ECMO was 4 days (IQR 1–7) in patients with COVID-19, which was longer than in patients with influenza or bacterial pneumonia. Prone positioning before ECMO was more common in COVID-19 patients (83%) compared to those with influenza (78%) or other etiologies (58%). Conversely, vasopressor use before ECMO was more frequent in influenza patients (62%). RRT before ECMO was more common in COVID-19 patients (27%) compared to those with influenza (10%) or other causes (11%).

### French deprivation index distribution

**Fig. 1:**
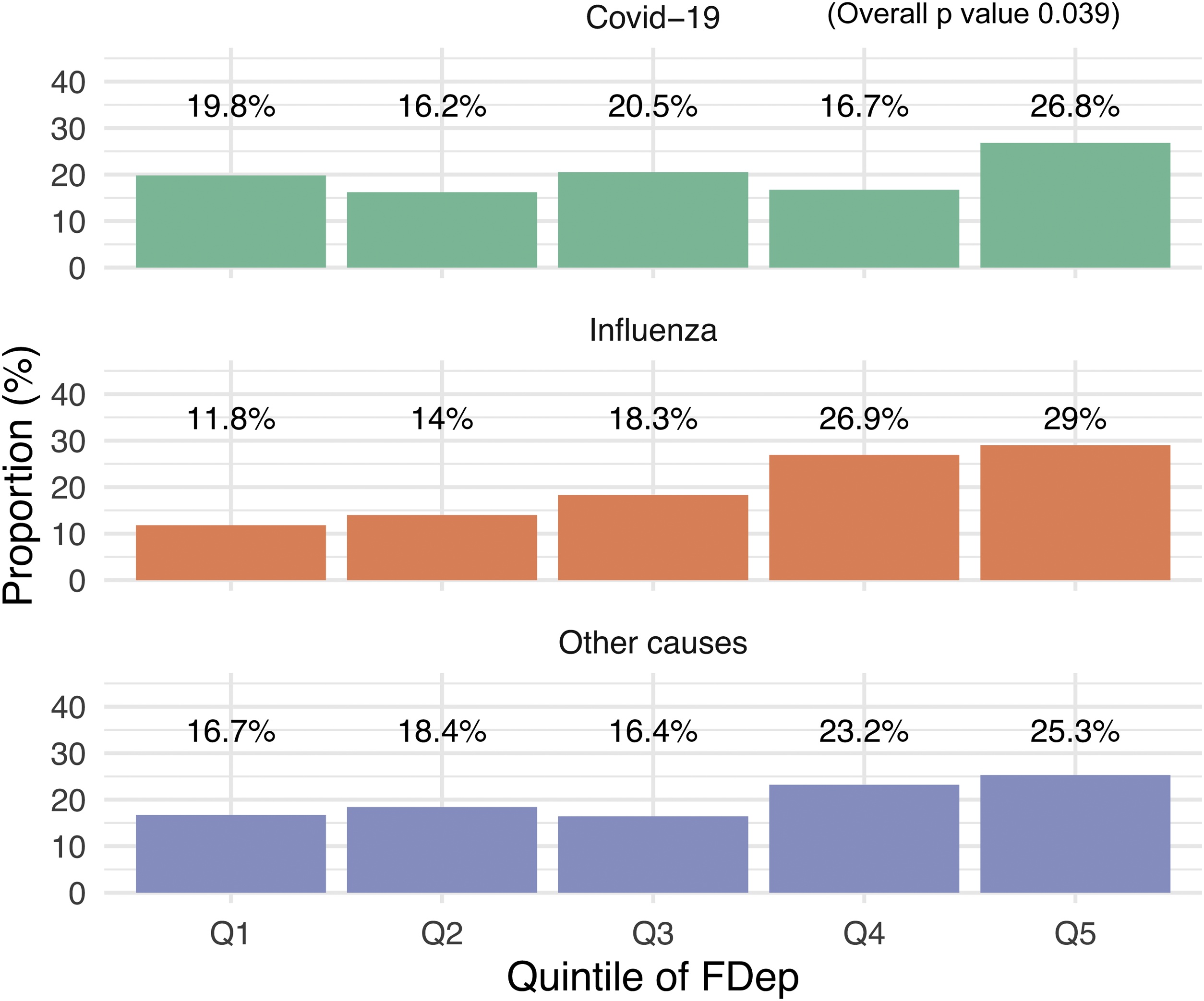
Distribution of FDep quintiles among ECMO patients according to the acute respiratory distress syndrome etiologies.

**Fig. 2:**
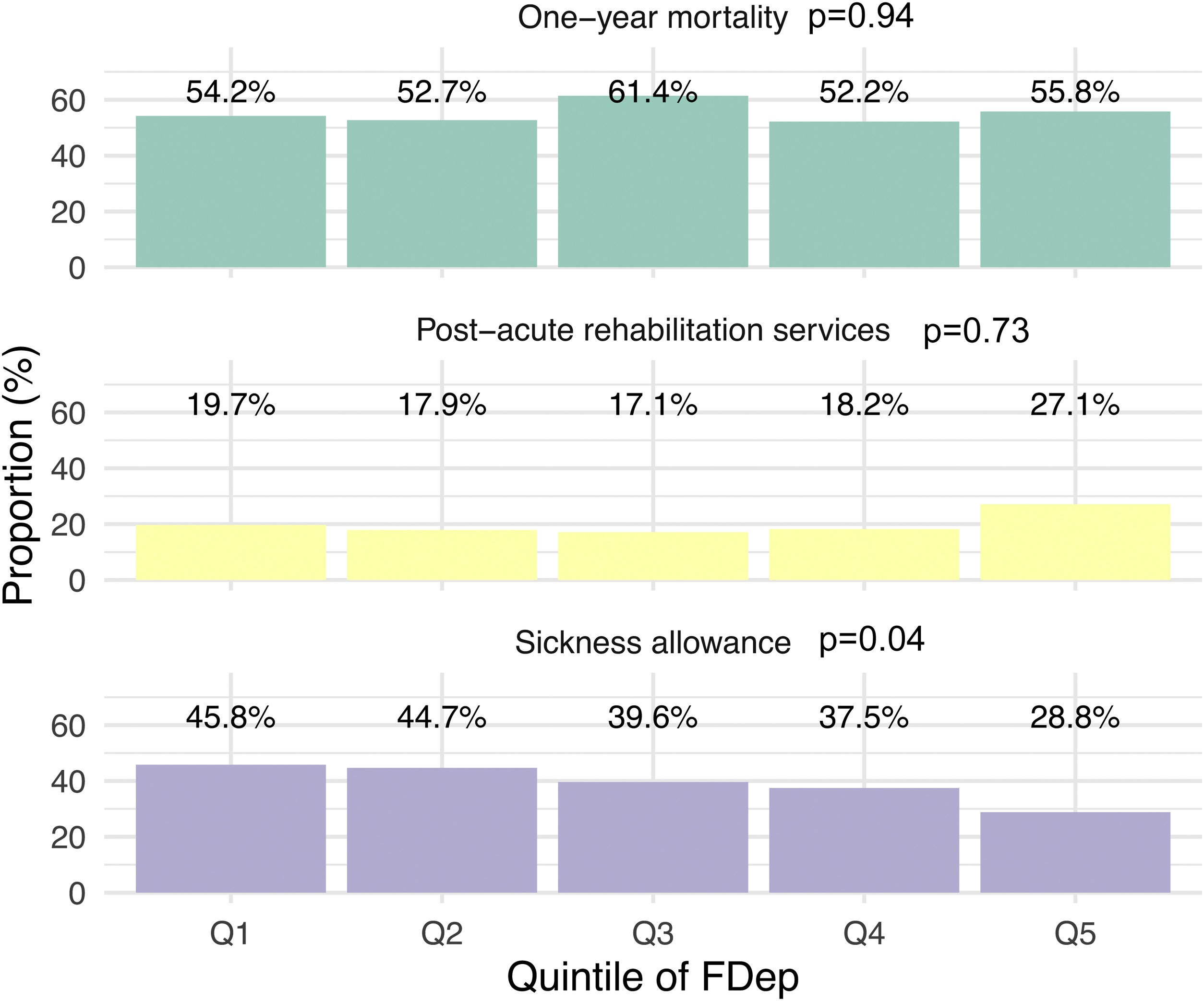
One-year mortality rate, rate of sickness allowance among survivors under 65 years, and rate of post-acute care transfers among survivors, depending on FDep quintile.

Among patients receiving ECMO, 27% resided in the most socioeconomically deprived areas, corresponding to the fifth quintile of the FDep (Fig. 1). This overrepresentation was observed across all ARDS etiologies: 29% of influenza patients, 26.8% of COVID-19 patients, and 25.3% of patients with ARDS from other causes lived in the most deprived areas (FDep quintile 5) (p = 0.039). Conversely, only 11.8% of influenza patients and 19.8% of COVID-19 patients supported with ECMO resided in the least deprived (most advantaged) neighborhoods (FDep quintile 1) (p = 0.039). Notably, patients in the COVID-19 cohort were significantly less likely to live in socioeconomically deprived areas (FDep quintiles 4–5) compared with those with influenza (33% vs. 56%) or ARDS from other causes (48%, p < 0.001 Table 1). Among survivors, individuals from the most deprived areas were less likely to receive sickness allowances (p = 0.040) during the year following hospital discharge, while their one-year mortality and use of post-acute rehabilitation services were similar to others (Fig. 2).

### In hospital outcomes and factors associated with in-hospital mortality

In-hospital mortality was 60% among patients with non-COVID-19 or non-influenza causes (60%), 56% in those with COVID-19, and 48% with influenza (p = 0.080). Among hospital survivors, COVID-19 patients had the longest median length of stay (56 [36–78] days), compared to patients with influenza (45 [32–64] days) and those with non-COVID-19 or influenza causes (46 [27–66] days). Similarly, durations of IMV and ECMO support were extended in the COVID-19 group relative to the other two cohorts (Table 2).

**Table 2:** Outcomes.

	ECMO for COVID-19 (N = 1245)	ECMO for influenza (N = 107)	ECMO for other causes (N = 370)	P-value
IMV duration, days	34 (20−54)	22 (11−37)	18 (10−31)	<.001
in hospital survivors	48 (30−68)	36 (26−52)	30 (20−51)	<.001
ECMO duration, days	13 (5−26)	9 (4−17)	6 (2−11)	<.001
in hospital survivors	13 (6−23)	10 (4−15)	7 (3−11)	<.001
ICU length of stay, days	37 (23−58)	28 (13−44)	24 (13−38)	<.001
in hospital survivors	48 (30−68)	36 (26−52)	30 (20−51)	<.001
Hospital length of stay, days	40 (26−63)	33 (17−51)	28 (15−50)	<.001
in hospital survivors	56 (36−78)	45 (32−64)	46 (27−66)	<.001
In-hospital mortality	698 (56)	51 (48)	221 (60)	0.080
One-year mortality	719 (58)	52 (49)	236 (64)	0.012

ECMO, extracorporeal membrane oxygenation; IMV, invasive mechanical ventilation; ICU, intensive care unit.

Data are presented as median (interquartile range) or N (%).

Multivariable analysis identified several independent predictors of in-hospital mortality, as summarized in Table 3. Increasing age was significantly associated with higher mortality, with patients older than 57 years showing the greatest risk (OR 3.55; 95% CI, 2.73–4.61). The requirement for RRT before ECMO initiation was also linked to increased mortality (OR 2.80; 95% CI, 2.11–3.72). After adjusting for these factors, COVID-19 was not independently associated with higher mortality compared to influenza, which served as the reference group. In contrast, non-viral causes of ARDS (i.e ECMO for other causes) were significantly associated with an increased risk of death (OR 1.70; 95% CI, 1.03–2.81). Notably, residing in the most deprived areas (FDep quintile 4−5) was not associated with an increase in in-hospital mortality (OR 0.91; 95% CI, 0.73–1.33). Similarly, being beneficiaries of the CMU-C was not associated with an increased mortality when FDep was introduced in the model in replacement of FDep (see eFile 3).

**Table 3:** Logistic regression modelling the risk of hospital death among patients who received ECMO*.

	Odds ratio (95% confidence interval)
Cause of ARDS	
Influenza	–
Covid-19	1.30 (0.82–2.05)
Other	1.70 (1.03–2.81)
Age	
⩽ 48 years	–
49−56 years	1.61 (1.18–2.20)
57 years and more	3.55 (2.73–4.61)
FDep quintile 4−5	0.91 (0.73–1.33)
Male	1.17 (0.92–1.48)
IMV-ECMO timing	
< 3 days	–
3−7 days	1.17 (0.89–1.55)
> 7 days	1.05 (0.79–1.40)
Prone positioning before ECMO	1.11 (0.84–1.47)
Vasopressors before ECMO	1.02 (0.82–1.28)
RRT before ECMO	2.80 (2.11–3.72)

ARDS, acute respiratory distress syndrome; ECMO, extracorporeal membrane oxygenation; FDep, French Deprivation Index Distribution; IMV, invasive mechanical ventilation; RRT, renal replacement therapy.

Assessment of collinearity in the model revealed no concerns, with all Variance Inflation Factor values within acceptable ranges. Model calibration was adequate, as reflected by a non-significant Hosmer–Lemeshow test (χ² = 12.6, df = 8, p = 0.13), indicating good agreement between predicted and observed outcomes. None of the evaluated interaction terms were statistically significant (all p > 0.05). The final model showed moderate discriminative ability, with a C-statistic of 0.688.

* Performed in 1460 patients.

### One-year outcomes

One-year mortality was higher in patients with bacterial pneumonia or other causes (64%) and COVID-19 (58%) compared to those with influenza (49%) (p = 0.012). Transfers to post-acute care facilities within one month of discharge occurred more frequently in COVID-19 patients (62%) than in those with influenza (46%) or ARDS from other causes than COVID or influenza (39%). In the year following discharge, 24% of COVID-19 survivors required long-term oxygen therapy. They were also more frequently rehospitalized (55%) than patients with influenza (43%) or ARDS caused by other reasons (34%). Among individuals younger than 65 years, the likelihood of receiving at least one day of sickness allowance during the first year after discharge was higher in the COVID-19 group (40%) compared with those with influenza (26%) or non-COVID and influenza ARDS (19%) (Table 4).

**Table 4:** One-year outcomes among ECMO patients discharged alive from the hospital.

	ECMO for COVID-19 (N = 547)	ECMO for influenza (N = 56)	ECMO for other causes (N = 149)	P-value
Post acute care and rehabilitation	339 (62)	26 (46)	58 (39)	<.001
Hospital at home service	11 (2)	2 (4)	1 (1)	0.347
Long-term oxygen therapy	129 (24)	6 (11)	25 (17)	0.026
Rehospitalization during the year	129 (55)	24 (43)	51 (34)	<.001
New consumption of beta-2 agonists	37 (3)	7 (7)	22 (7)	0.002
New pulmonologist consultation	251 (22)	24 (24)	39 (14)	0.016
Receiving least one sickness allowances in the year following hospital discharge*	199 (40)	12 (26)	23 (19)	<.001
Number of days of sickness allowances	135 (88−190)	173 (90−252)	82 (34−153)	0.018

ECMO, extracorporeal membrane oxygenation.

* Among people < 65 years old with paid employment before hospitalization in the ICU.

## Discussion

In this large nationwide cohort of 1722 patients who received ECMO for acute respiratory failure over 7 years, individuals from the most socioeconomically deprived areas were overrepresented. This pattern was consistent across different causes of ARDS. No crude difference in hospital mortality was observed among patients with COVID-19, influenza, or other causes. Independent factors associated with hospital death included older age and the need for RRT at the start of ECMO, while living in deprived areas was not associated with worse outcomes. After adjusting for these factors, mortality was higher in patients with non-COVID-19 and non-influenza causes. Notably, survivors who received ECMO for COVID-19 had longer durations of ECMO support and ICU stays, as well as more frequent complications. These patients also required more rehabilitation, long-term oxygen therapy, and experienced more rehospitalizations within the following year.

Since the start of the COVID-19 pandemic, mounting evidence has highlighted the disproportionate impact of social deprivation on health outcomes. People from deprived backgrounds have consistently shown higher rates of exposure [[18], [19], [20]], infection [18,21], hospitalization, and mortality [18,19,22]. These disparities are also evident in critical care. For instance, during the first wave of the COVID-19 pandemic, Lone et al. reported that among ICU admissions, 14% of patients came from the least deprived FDep quintile, whereas 25% were from the most socioeconomically disadvantaged areas. Patients in this latter group had a higher risk of 30-day mortality [23]. Likewise, Nordberg et al., using individual-level data, found that patients of African descent admitted to Swedish ICUs for COVID-19 who required IMV had a higher 90-day mortality risk, while higher income was linked to better survival [24]. Our study confirms these findings, demonstrating that the most severe cases of ARDS requiring ECMO disproportionately impact individuals from the most disadvantaged socioeconomic backgrounds. Importantly, this pattern was not specific to the COVID-19 pandemic. Similar trends were observed during the influenza pandemic and even in non-pandemic periods among patients receiving ECMO for other indications, with a substantial proportion belonging to the two most deprived socioeconomic quintiles. Moreover, we found that patients in the COVID-19 cohort were significantly less likely to reside in socioeconomically deprived neighborhoods compared with other etiologies. This finding may reflect selection effects driven by the intense hospital surge during the COVID-19 pandemic, which placed exceptional pressure on healthcare resources in France at that time. Several hypotheses may explain why severe ARDS requiring ECMO is more prevalent in deprived populations. Living conditions characterized by overcrowding may lead to higher exposure to viral loads, which is particularly relevant in the transmission of respiratory viruses such as SARS-CoV-2 and influenza [25]. Surprisingly, this trend was also observed among patients with less transmissible etiologies, such as non-COVID-19 and influenza etiologies, including bacterial pneumonia, suggesting that underlying comorbidities, such as hypertension and diabetes, which are more common in socioeconomically disadvantaged groups, may play a significant role in the development of severe ARDS [26].

Although disparities in healthcare access might explain such findings in some countries, this is unlikely in the French healthcare system. In our cohort, patients from the most socioeconomically deprived areas had similar access to post-acute rehabilitation services following discharge. A similar observation has been reported among less severe COVID-19 ARDS survivors, in whom socioeconomic status showed no significant impact on respiratory sequelae six months after ICU discharge [27,28]. Moreover, the organization of the ECMO network in France, including 24/7 mobile ECMO teams coordinated through specialized centers, has likely reduced regional disparities in access to this advanced therapy. Lower sickness allowance observed in these areas likely reflects higher unemployment rates, which prevent eligibility for such benefits. Reassuringly, despite the higher burden of severe ARDS, we found that living in a deprived area was not independently associated with higher mortality in our multivariable analysis.

Interestingly, although COVID-19 was associated with greater healthcare resource use and a higher incidence of ICU complications, it did not translate into worse in-hospital outcomes. In contrast, ARDS due to non–COVID-19 and non-influenza etiologies was independently associated with increased in-hospital mortality. Notably, nearly one quarter of COVID-19 survivors required long-term oxygen therapy, a markedly high proportion that was significantly greater than in both the influenza and other-cause groups. This incidence also exceeds previously reported rates for long-term outcomes in non–COVID-19 ARDS supported with ECMO [29] and for ARDS not treated with ECMO [30]. Age proved to be a crucial prognostic factor, a finding that aligns with previous studies involving patients on ECMO for COVID-19, influenza, and other causes [[31], [32], [33]]. The timing of ECMO initiation relative to mechanical ventilation is a well-recognized determinant of outcomes [31,32], but was not independently associated with mortality in our model. Although the interval from non-invasive support initiation to VV-ECMO may represent an even stronger predictor of mortality [34,35], the duration from intubation to ECMO remains clinically relevant. No single threshold should serve as an absolute contraindication, but prolonged delays are associated with increased risk and should be carefully evaluated on a case-by-case basis. During the pandemic, this interval was frequently prolonged due to the widespread use of prone positioning, despite prior evidence showing a substantial increase in mortality when ECMO is initiated beyond 7 days of IMV [31,32]. Finally, our findings underscore the critical prognostic impact of kidney failure in this setting, consistent with observations in both COVID-19– [15,35] and non–COVID-19– related ARDS requiring ECMO [[31], [32], [33]].

This study has several limitations. First, it spans a seven-year period, during which ECMO practices and clinical experience likely evolved. Notably, the COVID-19 pandemic introduced additional variability, particularly due to frequent shortages of ICU beds and critical care resources. Second, information on the ECMO experience or case volume of participating centers was not available. Given that center volume is strongly associated with patient outcomes [36,37], the relatively high in-hospital mortality observed across all three groups (>50%), substantially higher than in trials or cohorts conducted in high-volume expert centers [38,39], may be partially attributed to this factor. Third, we used the FDep index as a proxy for socioeconomic deprivation. While it offers valuable information on the socioeconomic characteristics of residential areas, it does not fully reflect individual-level social inequalities. As a result, this may have obscured a potential association between socioeconomic status and outcomes. Lastly, our analysis relied on data from an administrative healthcare database, which, although comprehensive, lacks detailed clinical information. Consequently, some potential confounders, such as body mass index, could not be accounted for in our multivariable model. This represents an important limitation, particularly because obesity is expected to be overrepresented among patients with COVID-19.

## Conclusion

In conclusion, our findings show that the disproportionate burden of severe ARDS requiring ECMO among patients from socioeconomically deprived areas extends beyond COVID-19 to include influenza-related ARDS and other causes. Importantly, despite this increased exposure, socioeconomic deprivation was not linked to worse outcomes. These results highlight the critical need for equitable access to advanced therapies through strong, 24/7 mobile ECMO networks operating within a hub-and-spoke system. Lastly, the factors behind the higher incidence of severe ARDS in deprived populations need further investigation to guide future public health strategies.

## CRediT authorship contribution statement

DN, AK, and MS designed the study, collected the data, performed statistical analysis, interpreted the results, and wrote the manuscript. All authors read and approved the final manuscript.

## Consent for publication

According to French law, written informed consent was not required for this type of study. An internet page informs the public about the reuse of the database and their rights according to the European General Data Protection Regulation n° UE 2016/679 dated 27 April 2016 [11].

## Ethics approval and consent to participate

The SNDS database was created by French law n°2016-41 dated 26 January 2016 [40]. The purpose of the database is to facilitate research by reusing claim data after names and social security numbers have been removed. Condition of use and security applying to the database is defined by French government regulation dated 22 March 2017 [10]. As part of its public statistics missions, the Department for Research, Studies, Assessment, and Statistics (DREES) of the French Ministry of Health, has permanent access to the SNDS database.

## Funding

There was no funding for this study.

## Data availability

The data that support the findings of this study are available from the Department for Research, Studies, Assessment, and Statistics (DREES) of the French Ministry of Health but restrictions apply to the availability of these data and so are not publicly available.

## Declaration of competing interest

Matthieu Schmidt reports lecture fees from Getinge, Drager, and Xenios outside the submitted work. Alain Combes reports grants from Getinge and personal fees from Getinge, Baxter, and Xenios outside the submitted work.

All other authors report no competing interests.
