# Supplementary material for: Socioeconomic status and ECMO outcomes in severe ARDS
Source: Ann Intensive Care. 2026 Jan 16;16:100012. doi: 10.1016/j.aicoj.2025.100012 (PMC12934413; doi:10.1016/j.aicoj.2025.100012)
Supplement: Supplementary file 1 [file mmc1.docx]

**eFile 1 : ICD-10 codes used to identify comorbidities**

| **Comorbidities** | **ICD10 code** |
| --- | --- |
| Immunocompromised status |  |
| Solid organ transplantation | ‘Z94’ |
| Agranulocytosis | ‘D70’ |
| HIV infection | 'B20' 'B21' 'B22' 'B23' 'B24' |
| Medullary aplasia | 'D61' |
| Malignancy requiring chemotherapy | 'Z511' |
| Immunodeficiency codes | 'D80' 'D81' 'D82' 'D83' 'D84' |
|  |  |
| Chronic kidney disease | 'N18' 'N19' |
|  |  |
| Arterial hypertension | 'I10' 'I11' 'I12' 'I13' 'I14' 'I15' |
