# Supplementary figures and images for: Socioeconomic status and ECMO outcomes in severe ARDS
Source: Ann Intensive Care. 2026 Jan 16;16:100012. doi: 10.1016/j.aicoj.2025.100012 (PMC12934413; doi:10.1016/j.aicoj.2025.100012)

**eFile 2. Distribution of the FDep Across France at the Regional and Municipal Levels**


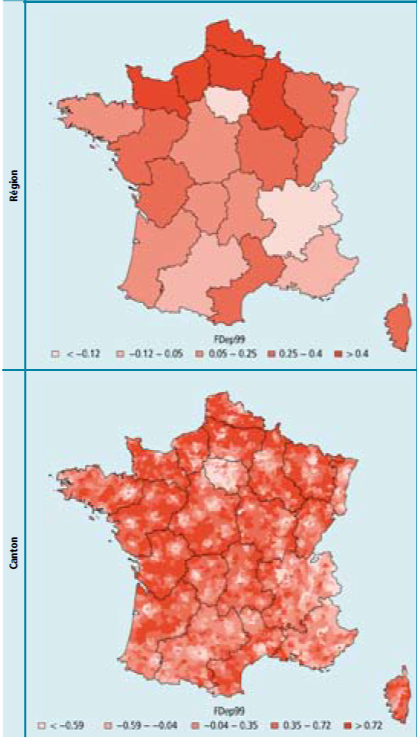

Supplement: Supplementary file 2 [file mmc2.docx]
