# Supplementary material for: Socioeconomic status and ECMO outcomes in severe ARDS
Source: Ann Intensive Care. 2026 Jan 16;16:100012. doi: 10.1016/j.aicoj.2025.100012 (PMC12934413; doi:10.1016/j.aicoj.2025.100012)
Supplement: Supplementary file 3 [file mmc3.docx]

**eFile 3: Logistic regression modelling the risk of hospital death among patients who received ECMO (with CMU-c as a personal surrogate of low income)**

|  | **Odds ratio (95% confidence interval)** |
| --- | --- |
| Cause of ARDS |  |
| Influenza | - |
| Covid-19 | 1.21 (0.78 – 1.87) |
| Other | 1.58 (0.98 - 2.53) |
| Age |  |
| ⩽ 48 years | - |
| 49-56 years | 1.64 (1.22 - 2.22) |
| 57 years and more | 3.53 (2.74 - 4.55) |
| CMUc benefeciary | 0.87 (0.66 - 1.16) |
| Male | 1.22 (0.97 - 1.53) |
| IMV-ECMO timing |  |
| < 3 days | - |
| 3-7 days | 1.18 (0.90 - 1.55) |
| > 7 days | 1.03 (0.78 - 1.36) |
| Prone positioning before ECMO | 1.07 (0.82 - 1.41) |
| Vasopressors before ECMO | 1.09 (0.88 - 1.36) |
| RRT before ECMO | 2.76 (2.10 - 3.64) |

*ARDS, acute respiratory distress syndrome; CMU-C, a French supplementary health insurance program providing free complementary coverage to individuals with very low income; ECMO, extracorporeal membrane oxygenation; FDep, French Deprivation Index Distribution; IMV, invasive mechanical ventilation; RRT, renal replacement therapy.*

*Model performed in 1567 patients (155 missing values)*
